# Role of Glia in Stress-Induced Enhancement and Impairment of Memory

**DOI:** 10.3389/fnint.2015.00063

**Published:** 2016-01-11

**Authors:** Jiah Pearson-Leary, Danielle Maria Osborne, Ewan C. McNay

**Affiliations:** ^1^Department of Anesthesiology and Critical Care Medicine, Children's Hospital of PhiladelphiaPhiladelphia, PA, USA; ^2^R.S. Dow Neurobiology Department, Legacy Research InstitutePortland, OR, USA; ^3^Behavioral Neuroscience and Biology, University at AlbanyAlbany, NY, USA

**Keywords:** glia, memory, stress, norepinephrine, hippocampus, glucocorticoids

## Abstract

Both acute and chronic stress profoundly affect hippocampally-dependent learning and memory: moderate stress generally enhances, while chronic or extreme stress can impair, neural and cognitive processes. Within the brain, stress elevates both norepinephrine and glucocorticoids, and both affect several genomic and signaling cascades responsible for modulating memory strength. Memories formed at times of stress can be extremely strong, yet stress can also impair memory to the point of amnesia. Often overlooked in consideration of the impact of stress on cognitive processes, and specifically memory, is the important contribution of glia as a target for stress-induced changes. Astrocytes, microglia, and oligodendrocytes all have unique contributions to learning and memory. Furthermore, these three types of glia express receptors for both norepinephrine and glucocorticoids and are hence immediate targets of stress hormone actions. It is becoming increasingly clear that inflammatory cytokines and immunomodulatory molecules released by glia during stress may promote many of the behavioral effects of acute and chronic stress. In this review, the role of traditional genomic and rapid hormonal mechanisms working in concert with glia to affect stress-induced learning and memory will be emphasized.

## Introduction

**Figure 1:**
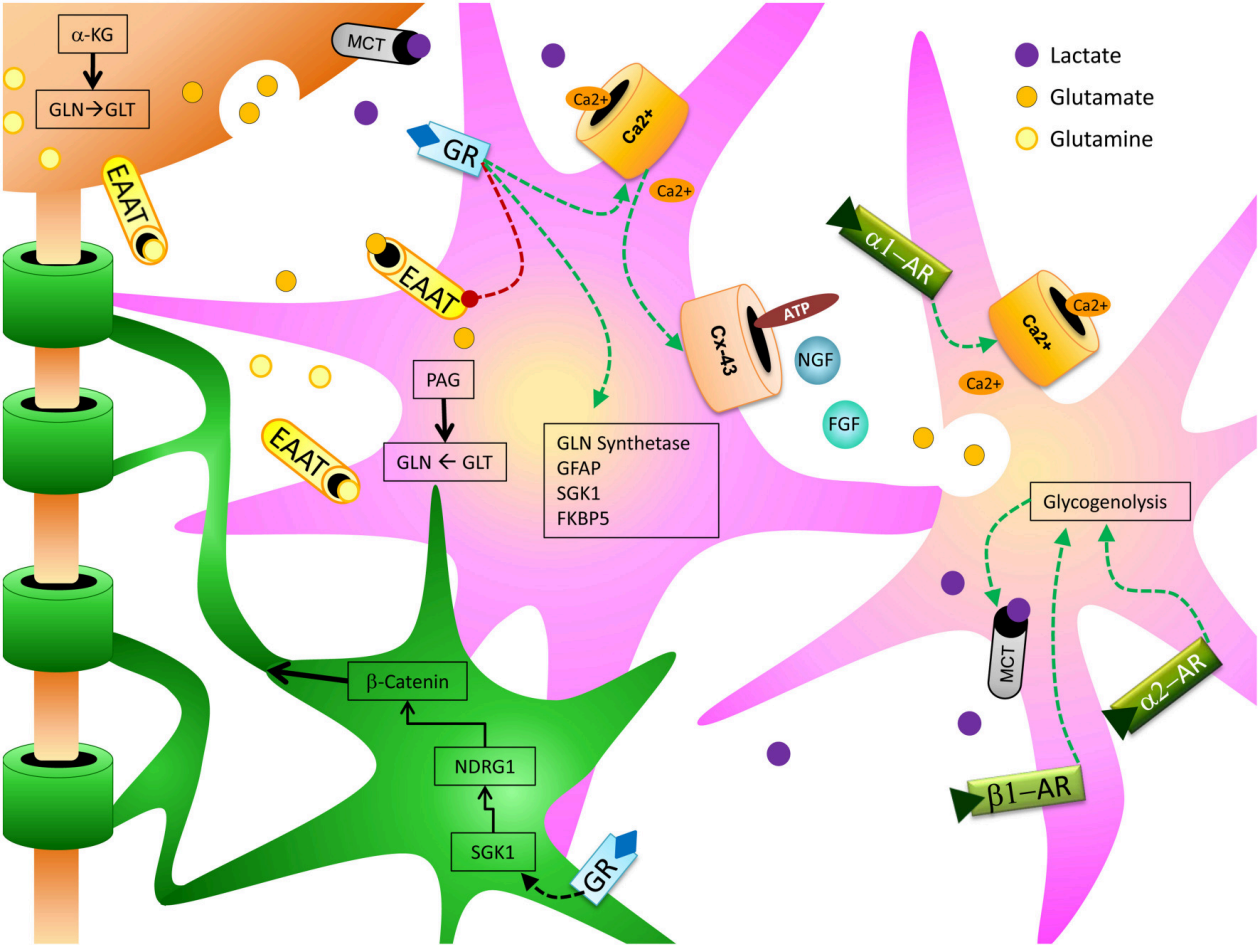
**Depicts the relationship between stress hormone effects on astrocytes (pink) and oligodendrocytes (green) and how they can support and enhance neuronal (orange) function to produce cognitive enhancing effects**.

Over the past several years, consensus on the role of glia in cognitive function has shifted from viewing them as primarily supportive of neuronal actions to recognizing them as critical players in neural function and behaviors such as learning and memory (Fields et al., 2014) (Figure 1). Astrocytes exert a variety of influences on neural processes critical for memory, including regulation of extracellular K^+^ concentration, provision of metabolic support (i.e., glucose and/or lactate) to neurons, and recycling of glutamate and GABA (Moraga-Amaro et al., 2014). Ramified (previously known as “resting”) microglia can control synaptic plasticity through release of several different cytokines that can modulate memory (Morris et al., 2013). An expanding field of research demonstrates that other types of glia, such as oligodendrocytes, can also influence processes underlying learning and memory (Fields et al., 2014). Conversely, stress can have a profound impact on glial structure and function (particularly astrocytes and microglia), which may affect the glial contribution to learning and memory. The impact of stress and stress-associated molecules, such as glucocorticoids (GCs) and norepinephrine (NE), on glial function has been the target of substantial investigation (Jauregui-Huerta et al., 2010). Many of the effects that glia exert on memory following stress may be mediated through neuroinflammatory processes (Frank et al., 2012), and the interaction between glia and stress hormones is likely to be bidirectional: many neuroimmune molecules released by glia, such as interleukin-1 (IL-1) and IL-6, can activate the hypothalamic-pituitary-adrenal axis (HPA) (Dinan and Cryan, 2012).

This review focuses on the specifics of interactions between stress hormones and glia, and the impact that these interactions have on learning and memory primarily via actions in the hippocampus.

## Stress axis overview

The HPA axis is the canonical regulatory system for stress hormones. Immediately following exposure to a stressor, epinephrine is secreted from the adrenal medulla and triggers a central stress response via stimulation of NE release from the locus coeruleus (LC) to multiple areas of the brain, including the hippocampus (Womble et al., 1980; Wong et al., 2012). NE in the paraventricular nucleus (PVN) then prompts the release of corticotropin releasing hormone (CRH). CRH and vasopressin promote secretion of adrenocorticotropic hormone (ACTH) from the anterior pituitary via pro-opiomelanocortin (Petrov et al., 1993). ACTH initiates the synthesis of GCs for release by the adrenal cortex. Whereas NE acts rapidly in the brain following release from the LC, GCs do not typically reach the brain for up to 10 min (Barbaccia et al., 2001), at which point they exert both signal transduction- and genomic-based effects in the brain (Salehi et al., 2010); these include feedback signaling in the hippocampus and other brain regions to negatively regulate the HPA axis and hence limit the neural impact of stress (Liberzon et al., 1999).

## Importance of stress to learning and memory

Acute stress can facilitate formation of highly salient and long-lasting memories, which in extreme cases may form the basis of post-traumatic stress disorder (Oitzl and de Kloet, 1992; Sandi and Rose, 1994a,b, 1997; Roozendaal et al., 1996; Sandi et al., 1997; Oitzl et al., 2001; Lupien et al., 2002a,b; Cahill and Alkire, 2003; Cahill et al., 2003). Increases in NE and GCs, either independently or to a greater extent together, act to increase integration of input from several regions [e.g., basal lateral amygdala (BLA), LC, cortex, PVN] whose outputs directly or indirectly converge on the hippocampus (de Kloet et al., 2005). Released early in the stress response, NE actions in the BLA are particularly important for enhanced memory consolidation following exposure to acute stress (Ferry and McGaugh, 1999; Hatfield and McGaugh, 1999; LaLumiere et al., 2003; Lalumiere and McGaugh, 2005). BLA lesions attenuate the procognitive effects of NE (Liang and McGaugh, 1983; Liang et al., 1990; Roozendaal and McGaugh, 1996), and administration of adrenergic antagonists, particularly β-adrenergic receptor (AR) antagonists to the amygdala (i) produces similar attenuation of stress enhanced memory and (ii) prevents GC-mediated increases in hippocampal-dependent learning (Liang et al., 1986; Quirarte et al., 1997; Roozendaal et al., 1999). Compared to the actions of NE in the BLA, direct effects of NE in the hippocampus are not as clear, although infusions of NE to the hippocampus can improve contextual fear learning (Yang and Liang, 2014). Again these effects appear to be dependent on β-AR activation, as propranolol administration prior to or following training diminished contextual fear performance (Stuchlik et al., 2009; Kabitzke et al., 2011).

The effects of elevated GCs on learning and memory are more complicated and time/dose sensitive than those of NE. Both mineralocorticoid (MRs) and glucocorticoid receptors (GRs) are present throughout the brain, and the hippocampus has the highest level of receptor co-localization (Sarrieau et al., 1984; Reul and de Kloet, 1985, 1986; Van Eekelen et al., 1988; Herman et al., 1989; Decavel and Van den Pol, 1990; Funder, 1994; Cullinan, 2000; Reul et al., 2000a,b; Barbaccia et al., 2001); consistent with this, GCs are potent modulators of hippocampal memory processes. Stress-mediated rises in GC levels following learning can improve memory formation, but proximate to recall GCs may impair memory retrieval (Oitzl and de Kloet, 1992; Kirschbaum et al., 1996; Sandi and Rose, 1997; Oitzl et al., 2001; Roozendaal, 2002; Joëls, 2006). GCs can also mask the mnemonic effects of NE when administered prior to NE (Borrell et al., 1984; Joels and de Kloet, 1989; Roozendaal, 2003; Richter-Levin, 2004). The effects of GCs on memory processing vary not only with the temporal relationship of increases in GCs to the event being remembered, but also according to the level of GC increase. The impact of GCs on the hippocampus (particularly CA1) follows an inverted-U-shaped dose-response curve (Joëls, 2006; Polman et al., 2013). Removal of GCs by adrenalectomy results in impaired consolidation, an effect that can be rescued by administering moderate doses of GCs (Joëls, 2006; Spanswick et al., 2011), so that low levels of GCs appear to mediate not only procognitive effects of moderate stress but also the formation of memories under baseline, non-stressed conditions. Moderate, physiological increases in GCs can improve cognitive processes, but very high elevations in GCs acutely impair hippocampal function (Salehi et al., 2010). Similarly, very high and/or prolonged GC exposure markedly impairs subsequent hippocampal function with results including cognitive deficits, hippocampal atrophy, metabolic dysfunction, and central insulin resistance (Sapolsky et al., 1985; Sapolsky, 1996; Willi et al., 2002; Joëls et al., 2004; Stranahan et al., 2008; Karatsoreos et al., 2010; Yun et al., 2010; Ye et al., 2011; Reagan, 2012).

Astrocytes are the most widely studied glial cell-type with regard to both memory processes and the effects of stress. It has been known for decades that chronic stress is associated with a decrease in hippocampal and prefrontal cortex volume (Fuchs and Flügge, 2003). More recent studies have shown that much of the brain volume reduction caused by chronic stress is accounted for by a large decrease in astrocytes, rather than neurons (Rajkowska and Miguel-Hidalgo, 2007). Similar outcomes of stress, and of elevated GCs in particular, have been observed in animal models: short-term stress increasing astrocyte volume (as measured by GFAP immunoreactivity), while chronic stress decreases astrocyte volume (Lambert et al., 2000; Jauregui-Huerta et al., 2010).

## Glucocorticoid-specific astrocytic actions: stress and memory

Astrocytes can influence memory in a variety of ways including (i) control of glutamate reuptake, synthesis and metabolism, (ii) regulation of calcium dynamics, (iii) large-scale coordination of neural activity via release of gliotransmitters, (iv) regulation of blood flow and hence glucose supply, and (v) provision of lactate as a metabolic substrate for neurons (Sahlender et al., 2014). Both GRs and MRs are expressed by astrocytes, and GCs have potent effects on astrocytic function (Jauregui-Huerta et al., 2010). Hence, it follows that GC-mediated modulation of astrocyte activity would influence cognitive processes. Here, we will focus on the known impact of GCs on memory processes mediated by astrocytes.

Astrocytes play a key role in regulating glutamate metabolism and activity. Following neural release of glutamate into synapses, astrocytes can remove glutamate through glial-specific glutamate transporters and convert glutamate into glutamine. Glutamine can then be used as an energy substrate in astrocytes or exported to neurons, where it can be re-converted into glutamate (Schousboe et al., 1993). Many of the long-term effects of chronically elevated GCs have been linked to excitotoxicity; GC-mediated dysfunction in astrocytes may prevent optimal glutamate clearance and therefore promote excitotoxicity (Popoli et al., 2012). Moreover, GC-induced dysfunction of astrocytes may also affect calcium metabolism and regulation, which will also impair glutamate regulation and thus increase the risk of excitotoxicity. Indeed, an important candidate mechanism for transduction of mnemonic regulation by astrocytes is calcium signaling. Regulation of calcium release and sequestration is also affected by GCs and stress. In both neurons and astrocytes, GCs control calcium homeostasis and signaling (Simard et al., 1999; Chameau et al., 2007; Suwanjang et al., 2013). Activated GRs can increase mitochondrial buffering capacity, leading to a reduction in cytosolic calcium (Psarra and Sekeris, 2009), and GCs can increase astrocytic calcium waves (Simard et al., 1999). These effects feed back into regulation of glutamate, discussed above: astrocytic calcium signaling controls release of glutamate (Volterra and Meldolesi, 2005). Calcium influx in astrocytes promotes release of gliotransmitters, which can include amino and nucleic acids, ATP, growth factors, glutamate, and/or peptides (Parpura et al., 2010, 2011). Gliotransmitters have been associated with regulation of the multipartite synapse, where they regulate neural excitability and can hence modulate memory processing (Hassanpoor et al., 2014). Inhibition of gliotransmitter release by blocking Cx-43 hemichannels (which release gliotransmitters) in astrocytes prevents the formation of long-term fear memories (Stehberg et al., 2012).

Stress can affect a variety of growth factors involved in learning and memory. For example, nerve growth factor (NGF) and fibroblast growth factor (FGF) are both increased following exposure to GCs or stress (Mocchetti et al., 1996; Molteni et al., 2001; Gubba et al., 2004; Chang et al., 2005; Kirby et al., 2013; Hashikawa et al., 2015). FGF in particular is critical for homeostatic regulation of astrocytes, and changes in FGF are largely responsible for the transition of astrocytes from a nonreactive to a reactive state (Kang et al., 2014a,b). These findings are intriguing because NE signaling can increase astrocyte reactivity (Griffith and Sutin, 1996); thus suppression of astrocyte reactivity by GC-induced release of FGF may moderate increased overall astrocyte reactivity following prolonged sympathetic activation. NGF has many roles, not limited to effects on glia; these include increasing both survival and differentiation of newly-maturing neurons and supporting hippocampal-dependent memory processes and cholinergic signaling (Chao, 2003; Mufson et al., 2003; Capsoni and Cattaneo, 2006; Schindowski et al., 2008; Aboulkassim et al., 2011).

GRs in the nucleus act as a transcription factor. Because different cell types have unique transcriptomes, it is worth discussing how GRs specifically influence gene transcription in astrocytes. In mature astrocytes GCs have been found to regulate a specific subset of genes that differs from that regulated in neurons. Early work demonstrated that transcription of glutamine synthetase and glial fibrillary acidic protein, both astrocyte-specific, is under GC-mediated control (Nichols et al., 1990; Laping et al., 1994), suggesting that both metabolic function and morphology of astrocytes can be influenced by GCs at the level of mRNA. Of the numerous genes affected by GCs in hippocampal astrocytes, some stand out as playing a prominent role in cognition and are under differential control by GCs based on dose/duration of exposure (Carter et al., 2013). Acute exposure to GCs, at doses sufficient to activate GRs, results in significantly increased astrocytic mRNA levels of adenosine 2b receptor, FK506 binding protein (FKBP5), pyruvate dehydrogenase kinase 4, and serum/glucocorticoid-inducible kinase-1 (Sgk1), while significantly decreasing early growth response protein 2 (Egr2) and wingless-related MMTV integration site 7a (Wnt7a). In contrast, chronic exposure to GCs produced effects that in some cases are opposite of the acute effects. Chronic GCs decrease hippocampal RNA expression of growth associated protein 43 (Gap43), histone deacetylase 7 (Hdac7), and synapsin II. Additionally, chronically elevated GCs decrease adenosine receptor 2b and Sgk1, while the effects of acute vs. chronic are the same for FKBP5 and Wnt7a. Similar effects were observed in the cortex, demonstrating that acute and chronic exposure to GCs have different effects on astrocytic gene expression (Carter et al., 2012, 2013) including that of several genes whose products are important in memory processing: in general, these changes are consistent with enhancement of memory processing after acute elevation in GCs but impairment after chronic GC elevation. Moreover, prolonged elevations in GCs can lead to a reduced number of astrocytes (Unemura et al., 2012). Given the data discussed in this review showing the importance of astrocytes in mediating neural processes critical for memory, it is likely that changes in gene expression and a reduction in astrocyte quantity and function following prolonged stress and GC exposure may underlie some of the cognitive deficits observed following long-term stress.

As noted, many of the astrocytic genes changed by GCs have products that are involved in memory processing. FKBP5 is particularly interesting as it can control stress reactivity (Schmidt et al., 2015). FKBP5 is a chaperone protein required to shuttle GRs to the nucleus (Binder, 2009). It has also been heavily implicated in early life stress programming, epigenetic regulation of stress responding (Klengel et al., 2013), and susceptibility to chronic stress later in life (Hartmann et al., 2012; Guidotti et al., 2013; Radley et al., 2013). Patients with PTSD have decreased FKBP5 expression, and successful cognitive-behavioral therapy in PTSD patients increases FKBP5 expression and hippocampal volume (Levy-Gigi et al., 2013). It is unclear, however, whether many of these effects of FKBP5 are mediated through glia or neurons. Sgk1 has an ever-growing body of evidence to indicate that it is integral to GC effects to enhance cognition, which includes actions to activate CREB and increase AMPAR and NMDAR receptors at the plasma membrane (Strutz-Seebohm et al., 2005; Yang et al., 2006; Lee et al., 2007; Tai et al., 2009; Lang et al., 2010). Currently, it is unclear as to whether the pro-cognitive activities of Sgk1 are mediated by neurons or astrocytes. Future research should examine the specific role of Sgk1 in astrocyte function given the robust increase in astrocytic Sgk1 following both acute and chronic GCs (Carter et al., 2013).

A further mechanism by which astrocytes may contribute to learning and memory is via metabolism of glucose leading to export of lactate, which will both regulate cerebral blood flow and potentially provide metabolic support to active neurons (Iadecola and Nedergaard, 2007). Some data suggest that acquisition of long-term fear memories may require astrocytically-derived lactate (Suzuki et al., 2011). Intriguingly, it is unknown as to what extent stress-induced molecules such as GCs can affect efficacy of lactate export from astrocytes. Astrocytes form a functional unit with neurons and blood vessels, which has been referred to as the neurovascular unit (Iadecola and Nedergaard, 2007). Many neuroinflammatory molecules that are released during stress are known to affect the neurovascular unit, and can increase “leakiness” of the blood-brain barrier (Kröll et al., 2009). In the amygdala, GCs produced by chronic stress can impair efficacy of the neurovascular unit by preventing vasodilation in response to neural activity (Longden et al., 2014). It is currently unknown whether neurovascular units in other brain regions respond similarly/differently to that of the amygdala, but such effects are likely to markedly impact cognitive processing. As a side note, such effects are also important to consider when interpreting fMRI or PET studies in which patients may be stressed, which is likely to be the case in the majority of such studies.

Overall, GCs exert a variety of effects on astrocytic function, from acute effects via calcium dynamics and release of gliotransmitters to transcription-mediated events, neurovascular control, and regulation of glutamate metabolism and glucose flux.

## Astrocyte-specific noradrenergic activities

Using rodents and chicks, respectively, Dr. Leif Hertz's and Dr. Marie Gibbs' groups have elucidated many roles for noradrenergic signaling in regulating astrocyte function and metabolism (Huang and Hertz, 1995; Hertz et al., 2007, 2010; Gibbs and Bowser, 2010). While most of this work has been aimed at unraveling cognitive aspects of astrocytic function under baseline conditions, these data can likely be extended to permit speculation on the impact of LC activation and subsequent NE release on cognitive function during stress.

Noradrenergic cell bodies are primarily located within the LC and project throughout the cerebral cortex, limbic system, and cerebellum (Swanson and Hartman, 1975), with prefrontal cortical and hippocampal regions receiving large amounts of noradrenergic innervation (Gibbs et al., 2010). A simplified primary function of LC-activation during stress may be to “boost brain power” and direct cognitive processes toward enhanced attention, improved vigilance, and a shift in memory processing toward retrieval of information relevant to the stressor (O'Donnell et al., 2012). NE may promote some of these cognitive effects via astrocytic release of glutamate, lactate production and transport to active neurons, and an increase in both glycogen metabolism and gliotransmitter release (O'Donnell et al., 2012; Moraga-Amaro et al., 2014): overall, one major effect is to increase glucose metabolism to meet the demands of cognitive processing. Hippocampal memory processes are well-established to be limited by glucose availability, and the procognitive effects of exogenous glucose administration have been suggested to be via glia rather than neurons (McNay and Gold, 2002).

NE acts via G-protein coupled receptors; α- and β- type 1 and 2 adrenergic receptors (ARs). Astrocytes primarily express β1, α1, and α2 (Hertz et al., 1984, 2010; Deecher et al., 1993). Like GCs, NE regulate astrocytic calcium signaling (Gibbs and Bowser, 2010): administration of the α1 agonist phenylephrine increases intracellular Ca^2+^, and similarly stimulation of the LC increases intracellular astrocytic Ca^2+^ via an α1-dependent mechanism (O'Donnell et al., 2012). Because NE activation during stress precedes GC activation, it is possible that the reduction in intracellular calcium and increased mitochondrial calcium buffering induced by GCs acts to control increases in intracellular Ca^2+^ caused by NE signaling to prevent overexcitation and/or apoptotic events.

NE also has effects on astrocytic metabolism, and effects of both α-receptor and β-receptor signaling on astrocytic metabolism have been documented (Subbarao and Hertz, 1990, 1991; Hutchinson et al., 2007, 2008; Gibbs et al., 2008). Activation of α2 receptors on astrocytes can both promote glycogen storage and increase astrocytic glycogen breakdown (Hertz et al., 2007). Astrocytic β-receptor-mediated glycogenolysis is more effective than α-receptor mediated glycogenolysis (Subbarao and Hertz, 1990). Astrocytic glycogenolysis is critical for glutamate cycling, as well as providing lactate that may be especially important as a rapidly-utilizable energy source during cognitive demand. Both α1- and α2 adrenergic signaling in astrocytes can increase oxidative metabolism including lactate production (Subbarao and Hertz, 1991). NE can also increase glutamine uptake in astrocytes, which is another important energy metabolite in astrocytes (Huang and Hertz, 1995). Because energy provision is a rate-limiting step in neural activity, the several actions of NE to increase metabolic support for both astrocytes and neurons are likely key to producing increased strength of memories formed at times of moderate stress (Osborne et al., 2015). The specificity of NE receptor subtypes found on astrocytes offers a potential target for therapies aimed at specifically modulating glial responses to stress, including treatments for stress-related disorders.

## Microglial impacts on cognition

**Figure 2:**
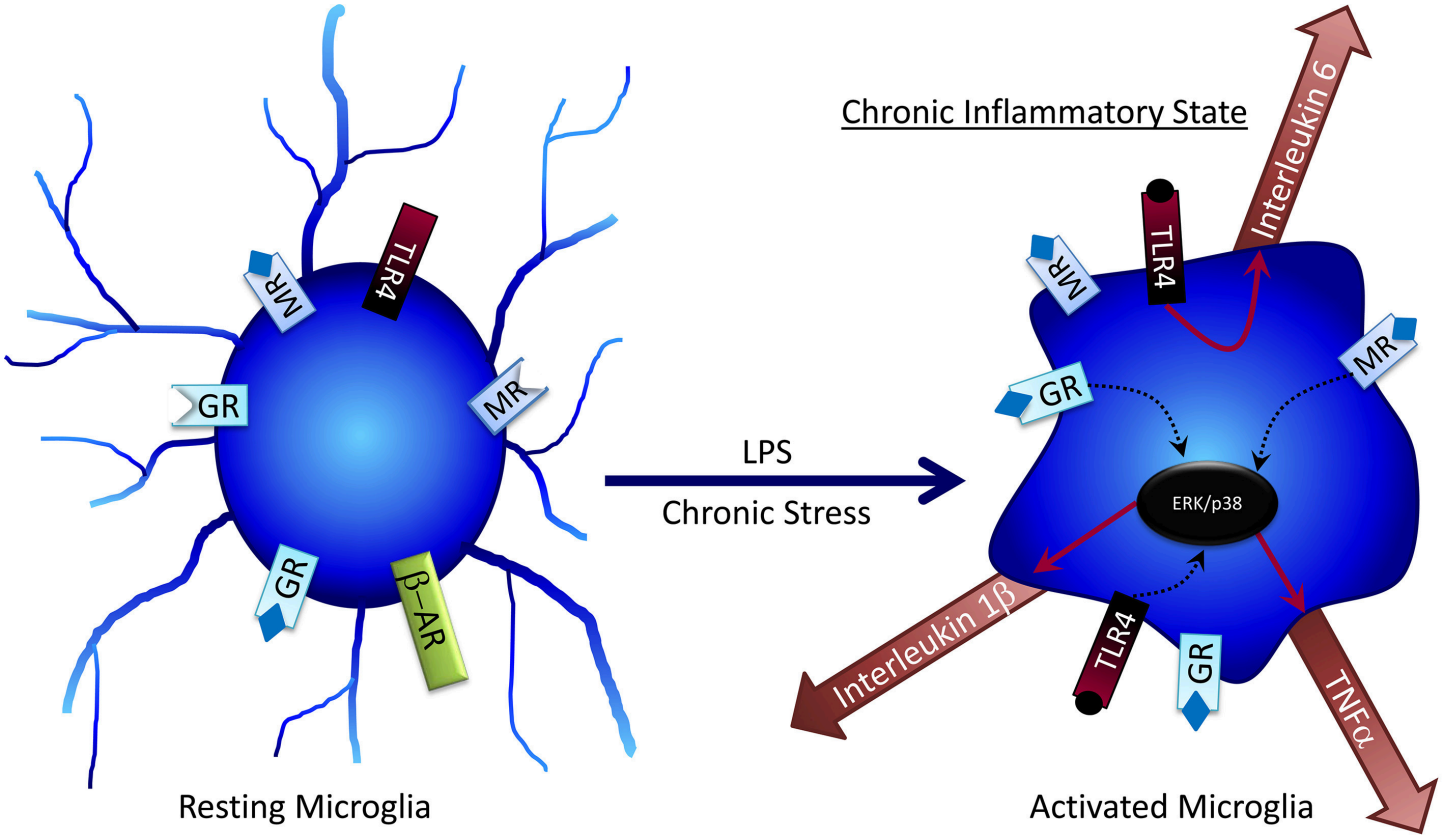
**Microglia can become activated by factors such as LPS or chronic stress, where they become desensitized to the anti-inflammatory effects of glucocorticoids**. Activated microglia can release pro-inflammatory cytokines, which at high concentrations can have negative effects on cognitive processes.

No longer regarded as solely an immune cell of the brain, several studies show that microglia are also involved in regulating neural activity (Thomas, 1992; Ilschner et al., 1996; Kettenmann, 2007), primarily through release of neuroimmune molecules such as cytokines that modulate surrounding neurons and astrocytes (Figure 2). Chronic unpredictable stress (CUS) promotes an increase in microglial proliferation and activation in a variety of brain regions including the hippocampus; however, following 5 weeks of CUS exposure microglial activity declines below baseline (Kreisel et al., 2014). Treatment with a variety of microglia-activating molecules such as endotoxin, macrophage colony-stimulating factor, or granulocyte-macrophage colony stimulating factor can prevent CUS-induced depressive behaviors (Kreisel et al., 2014), suggesting that microglia may be key regulators of adaptation to chronic stress.

Interleukin-1β (IL-1β) is perhaps the best-characterized cytokine released in response to stress. Following chronic mild stress, mice have impaired memory on both object location and object recognition memory. Cognitive impairments are accompanied by increased plasma IL-1β, plasma tumor necrosis factor-alpha, and IL-6 (Li et al., 2008). After chronic mild resident-defeat stress, a subset of rats that develop pro-depressive behaviors shows increased brain IL-β expression, and inhibition of brain IL-1β signaling prevents the pro-depressive symptoms (Wood et al., 2015). Independent of stress, studies have reported that IL-1β can impair memory, have no effect on memory, or enhance memory (Ross et al., 2003; Goshen et al., 2007; Huang and Sheng, 2010; Ben Menachem-Zidon et al., 2011; Bitzer-Quintero and González-Burgos, 2012; Pascual et al., 2012; Arisi, 2014; Jones et al., 2015), indicating that further work is needed.

Other microglial-released cytokines and immune molecules, such as IL-6 and TNF-α, can affect memory (Tonelli and Postolache, 2005; Nelson et al., 2013; Williamson and Bilbo, 2013; Arisi, 2014; Grinan-Ferre et al., 2015; Smith et al., 2015). In general, effects of cytokines are highly dependent on timing, dose, and duration; but the potentially confounding effects of inflammation can make interpretation difficult and are a major contributing factor to every major neurological disease (Bibi et al., 2014; Daulatzai, 2014; Legido and Katsetos, 2014; Nisticò et al., 2014; Stuart and Baune, 2014; Patterson, 2015; Walker and Lue, 2015). Chronic stress, poor diets, and acute traumatic stress can all induce elevated cytokine release, negatively affecting learning outcomes (Boitard et al., 2014; Hsu et al., 2015; Jones et al., 2015; Yazir et al., 2015).

Important to discuss, as a component of microglial effects following stress, is the kynurenine pathway (KP). Following chronic stress, tryptophan can be directed toward the KP (Miura et al., 2008), primarily driven by cytokine induction of indoleamine 2,3,-dioxygenase (IDO). In astrocytes, the KP increases production of the NMDA receptor agonist kynurenic acid; in microglia, the KP increases production of the NMDA receptor agonist quinolinic acid (Jo et al., 2015). Given the key role of NMDA receptors in hippocampal memory processing, it is not surprising that recent data suggests that activation of the KP can affect memory processes, and thus may be a novel pathway with importance for stress-related memory dysfunction (Heisler and O'Connor, 2015; Varga et al., 2015).

## Microglia and glucocorticoids

**Figure 3:**
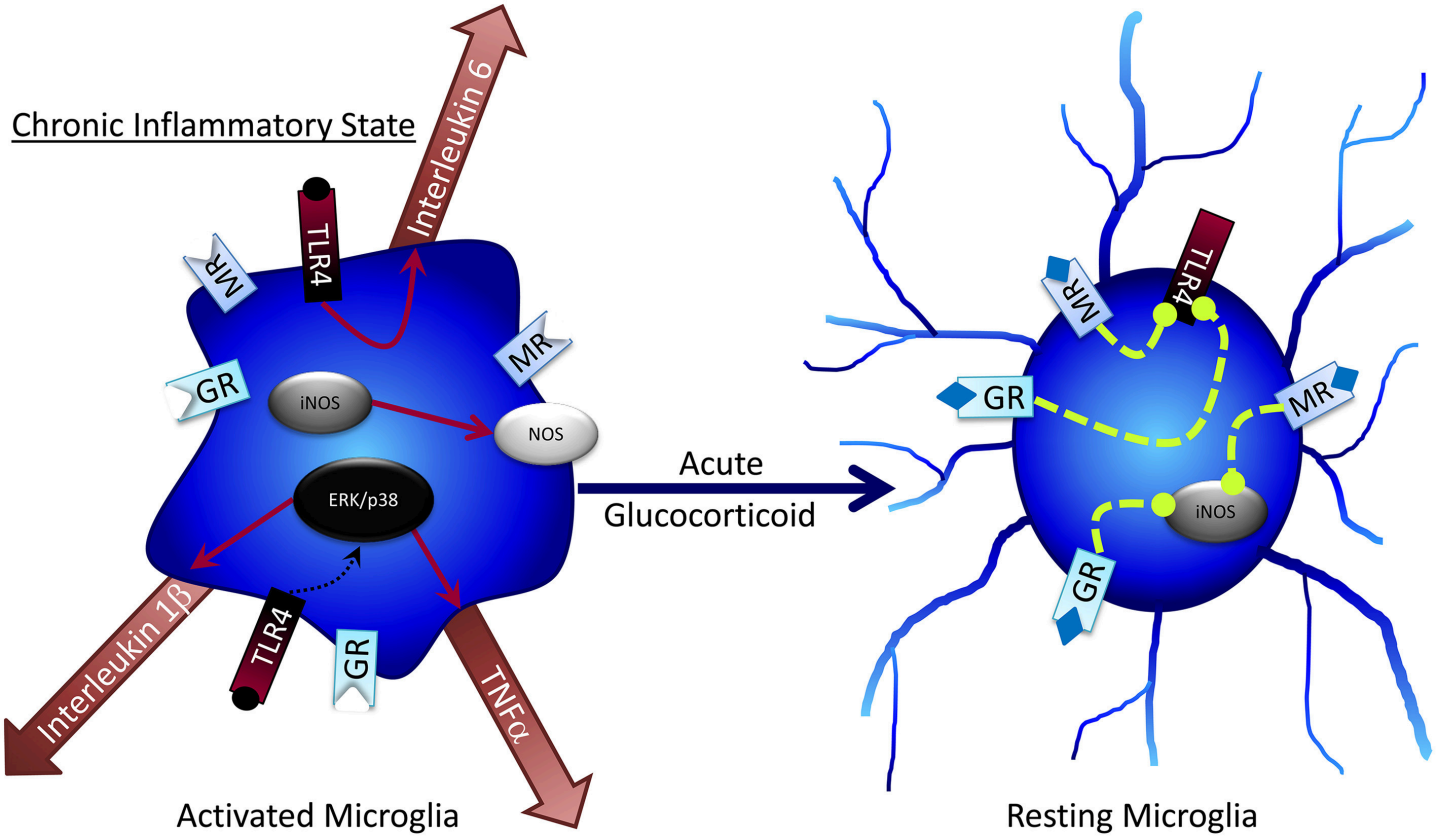
**Acute glucocorticoid exposure can return activated microglia to resting states**.

GCs have well-established anti-inflammatory effects that decrease microglial activation. GCs provide master control over several inflammatory and anti-inflammatory factors (Figure 3). Microglia express both MRs and GRs (Sierra et al., 2008), and GCs can suppress central inflammation through microglia (Goujon et al., 1996; Kawai and Akira, 2010). GC administration to microglial cultures suppresses nitric oxide release by blocking the expression of inducible nitric oxide synthase, which likely leads to a reduction in microglial-mediated cell death (Drew and Chavis, 2000). In mice lacking functional GRs on microglia, lipopolysaccharide treatment (LPS; a treatment that produces “active” microglia) can increase neuroinflammation and neural toxicity relative to wildtype mice treated with LPS (Carrillo-de Sauvage et al., 2013). In other studies, GR antagonism attenuated the effects of LPS-induced microglial activation including CA1 pyramidal cell loss, JNK and p38 activation and decreased Akt and CREB phosphorylation (Espinosa-Oliva et al., 2011); taken together, these findings strongly support a role for GCs in modulating microglia function, but suggest that the impact of GCs may—as with e.g., astrocytes—be critically dependent on dosage and timing.

Chronic stressors can attenuate or prevent the anti-inflammatory effects of GR activation on microglia. Microglia can readily become sensitized to GC over-secretion to the point where GCs no longer prevent pro-inflammatory microglial activity, but rather promote it, as is the case in neurodegenerative disease and obesity (Munhoz et al., 2006; Frank et al., 2012; Dey et al., 2014). Understanding of the links between diet-induced obesity, cognitive dysfunction, and neurodegenerative disease continues to expand and now encompasses a greater role for microglia in these events. Sustained GC release and impaired HPA negative feedback are hallmarks of obesity and Type 2 Diabetes (Bruehl et al., 2009; de Guia et al., 2014; Paredes and Ribeiro, 2014; Martocchia et al., 2015), cognitive dysfunction (McEwen and Sapolsky, 1995; Dumas et al., 2010) and Alzheimer's disease (Notarianni, 2013). These pathological conditions are also characterized by increased cytokine release, inflammation, and microglial activation (Xiang et al., 2006; Rodriguez et al., 2010; Buckman et al., 2014; Erion et al., 2014; Hwang et al., 2014; Heneka et al., 2015; Kälin et al., 2015; Lee et al., 2015; Ramos-Rodriguez et al., 2015).

## Microglia and norepinephrine

**Figure 4:**
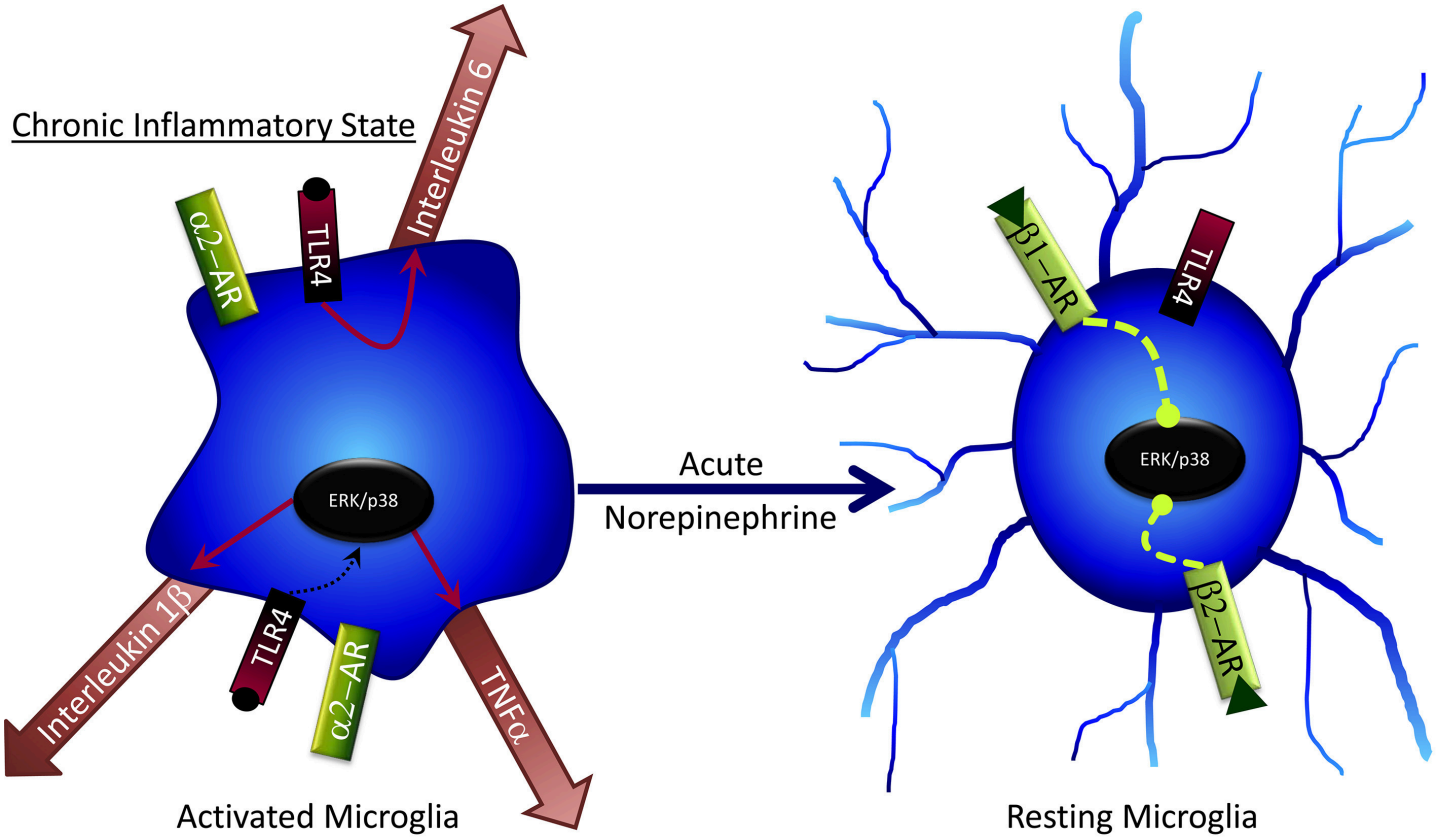
**Acute norepinephrine exposure can decrease release of cytokines by microglia via inhibition of ERK/p38 signaling**.

NE has several effects on microglia that may contribute to cognitive deficits following stress (Figure 4). Generally, NE is capable of subduing inflammatory response genes in microglia and astrocytes (Hetier et al., 1991; Feinstein et al., 2002; Tynan et al., 2012), such as the major histocompatibility complex (Frohman et al., 1988), IL-1β (Ballestas and Benveniste, 1997), and TNF-α (Ballestas and Benveniste, 1997; Tynan et al., 2012) via β-AR activation. While in a “resting” phase, microglia predominately express β2 and β1 ARs (Tanaka et al., 2002), but this can change to α2 expression following inflammatory activation (i.e., during stress). Bath application of NE to brain slices results in microglial process retraction and potential reversal of stress-induced inflammation (Mori et al., 2002). NE can also attenuate microglial process extension mediated by the gliotransmitter ATP (Gyoneva and Traynelis, 2013); conversely, loss of NE decreases microglial migration to sites of inflammation (Heneka et al., 2010), confirming a key role for NE in microglial regulation. NE administration to cultured rat microglial cells decreases mRNA expression of several pro-inflammatory cytokines including IL-6 and TNF-α (Mori et al., 2002). Although under normal, non-pathological conditions these pro-inflammatory factors can have important procognitive roles, in disease or chronic stress states their effects can become pathogenic and lead to impaired cognitive function and cell death. Therefore, NE may decrease pathogenic microglial action following stress (Heneka et al., 2010). Taken together with findings of GCs on inhibiting microglial activation, it is intriguing that both NE and GCs can suppress microglial activation amidst a milieu of pro-inflammatory stimuli (e.g., pro-inflammatory cytokines, increased BBB permeability, and immune cell influx into the brain) that occur following stress. These findings may suggest that NE and GCs, via glia, act in part as anti-inflammatory stimuli that prevent pathogenic inflammatory activity in the brain following stress; the impaired response to these hormones seen after chronic and/or very high stress may include diminished ability to protect against brain inflammation, with deleterious consequences.

## Oligodendrocytes and stress

While most attention in this field has been given to the impact of astrocytes and microglia on cognitive function, there is evidence that oligodendrocytes are also affected by stress and could modulate learning and memory. Oligodendrocytes' list of known functions extends beyond axon myelination, and now includes direct modulation of neuronal function (de Hoz and Simons, 2015).

Although stress and/or GC administration almost ubiquitously decreases neurogenesis (Anacker et al., 2013; Lehmann et al., 2013; Schoenfeld and Gould, 2013; Anacker, 2014; Chetty et al., 2014), stress has the opposite effect on oligodendrogenesis (Chetty et al., 2014). As with neurons, corticosterone increases activation of SGK1 in oligodendrocytes and subsequently induces abnormal morphological changes in arborization via NDRG1 and catenin signaling (Miyata et al., 2011). This increased arborization has been linked to increased depression-like behavior in stressed mice (Miyata et al., 2015). Other research suggests that GCs provide important survival signals that can aid oligodendrocyte and oligodendrocyte precursor survival against cytokine toxicity (Melcangi et al., 2000; Mann et al., 2008). Further investigation into the role oligodendrocytes play in cognition is required, both at baseline and after stress, but early evidence supports a vital function for this cell type in cognitive responses to stress.

## Conclusion

Glia are increasingly recognized as critical regulators of cognitive processes; understanding of the multiple cell types involved in behavioral regulation and the molecular processes involved continues to expand. Glia are quickly becoming pharmacologically-relevant cellular targets for treatments of a variety of psychiatric disorders (Koyama, 2015) and offer a potential opportunity to regulate neural and cognitive responses to stress including treatment of stress-induced behavioral disorders. Such approaches are likely to take advantage of the potentially increased specificity offered by modulation mechanisms unique to glia rather than also affecting neurons.

## Author contributions

JP-L, DO, and EM all planned, drafted, and edited the manuscript.

## Funding

Merlin Trust 2015 Excellence award to ECM; American Diabetes Association 7-12-BS-126 to ECM.

### Conflict of interest statement

The authors declare that the research was conducted in the absence of any commercial or financial relationships that could be construed as a potential conflict of interest. The reviewer Professor Leif Hertz and handling Editor Professor Ye Chen declared past collaboration and the handling Editor states that the process nevertheless met the standards of a fair and objective review.
